# The Response of Purslane (*Portulaca oleracea*) to Soil-Added Pb: Is It Suitable as a Potential Phytoremediation Species?

**DOI:** 10.3390/toxics11020153

**Published:** 2023-02-06

**Authors:** Georgios Thalassinos, Spyridon A. Petropoulos, Vasileios Antoniadis

**Affiliations:** Department of Agriculture Crop Production and Rural Environment, School of Agricultural Sciences, University of Thessaly, Fytokou Street, 384 46 Volos, Greece

**Keywords:** accumulator plant, soil contamination, lead uptake, nitrogen, phytoextraction, toxicity stress, heavy metals

## Abstract

Soils with high lead (Pb) levels can be decontaminated with the use of tolerant plants. Their effectiveness may be increased with added soil N due to boosted plant vigor, but such an agronomic practice has not been widely reported so far. In this work, purslane (*Portulaca oleracea*) was tested in a pot experiment as a potential phytoremediation species using soil spiked with Pb at doses of 0, 150, 300, 600, and 900 mg kg^−1^ (referred to as Pb(0), Pb(150), Pb(300), Pb(600), and Pb(900), respectively) with added N (referred to as N(1); at 300 kg N ha^−1^) and without added N (N(0)). We found that added Pb did not cause any adverse effects on plant growth (height, and aerial and root dry biomass) and physiological parameters, which were boosted with added N. Lead plant concentration and uptake significantly increased with added N, a finding that confirms our hypothesis. The number of necessary harvests of purslane in order to reduce soil Pb to half its initial concentration was also calculated and found to decrease with added N, being 131 at Pb(900)N(1). Although results indicate the potential of purslane as a phytoremediation species, further research is needed under real field conditions.

## 1. Introduction

Lead (Pb) is a toxic element; it is normally found in soils through lithogenic processes, but its content may increase due to various anthropogenic activities [1]. In many areas around the globe, Pb is the major contaminant, posing risks to plants grown in contaminated soils, and ultimately to human health [2], despite the fact that Pb is considered to be a relatively immobile element in soil; thus, its bioavailability may, falsely under some conditions, be expected to be limited. Phytoremediation is a nature-based solution of decontaminating an affected area with the use of a tolerant species, which can serve either as an accumulator of a particular metal or as an excluder, thus allowing crop cultivation without severe effects on plant growth and development. In the former case (phytoextraction), the process leads to the gradual cleanup of the soil through the absorption of high concentrations of a given metal from plants—plants must then be harvested, discarded, and treated carefully with incineration; in the latter case (phytostabilization), plants manage to exclude that particular metal from uptake, thus allowing its cultivation, and if the crop is of commercial interest, they can substitute other species which are less tolerant and allow the exploitation of contaminated soils [3]. Finally, phytodegradation refers to the degradation of toxic pollutants through mechanisms such as phytovolatilization (plants absorb the pollutants from the soil and release them in a volatile form to the air through respiration), rhizodegradation, or phytodegradation by itself [4].

Wild species, including several halophytic plants, are well known for their high ability to tolerate stressful conditions due to heavy metal toxicity [5,6]. These properties are associated with mechanisms that allow them to exclude, exude, or sequestrate toxic ions and avoid their toxic effects [7]. For example, *S. densiflora* and *S. maritima* have been suggested for the phytoremediation of soils polluted with various heavy metals such as As, Cu, Fe, Mn, Pb, and Zn [8], whereas various *Atriplex* species are well known for their hyperaccumulation properties of Ni, Cu, Pb, and Zn [9]. Moreover, Zaier et al. [10] and Zaier et al. [11] reported the high potential of *Sesuvium portulacastrum* to accumulate and phytoextract Pb from contaminated soils. However, despite the promising phytoremediative properties of wild species, growing conditions are pivotal for their response to heavy metal toxicity and the removal of contaminants from the soil, especially when other stressors are also involved [12].

Purslane (*Portulaca oleraceae*) is a wild edible plant that can be commercially cultivated in pristine soils and traded as it can be used as a gourmet raw or cooked salad. It has recently been tested as a potential species for use in phytoremediation if introduced to Cr(VI)-contaminated soils [13,14]. Moreover, the phytoremediation potential of the species has been reported by Kale et al. [15], who suggested that hydroponically grown purslane may tolerate a high accumulation of Cr through the accumulation of proline and enhanced capacity of scavenging radical oxygen species. According to Elshamy et al. [16], purslane plants may serve as phytoextraction and phytostabilization species in heavy-metal-contaminated farmlands from industrial effluents, especially when various associated wild plants are also considered in the phytoremediation process. Dwivedi et al. [17] also reported the significant hyperaccumulating properties of *Portulaca* species (e.g., *P. oleracea* and *P. tuberosa*), especially in the case of Pb which recorded the highest concentrations in *P. oleracea* roots, followed by the shoots and flowers of the plants. Similarly, Amer et al. [18] tested three endemic species of the Mediterranean, including *P. oleracea*, in a hydroponic system where a nutrient solution was spiked with different concentrations of Pb (5, 10, 25, 50, and 100 mg L^−1^) and suggested significant Pb contents in roots up to 10 mg L^−1^, whereas higher toxicity levels resulted in growth inhibition. The high capability of the species to tolerate heavy metal toxicity could be attributed to its ability to switch from C3 to C4 photosynthesis, which according to the literature may confer high tolerance to stressful conditions [19].

The phytoremediation potential of a given plant could be boosted if a beneficial factor contributes to increased plant growth and vigor, thus mitigating the negative effects of toxic element stress. For example, Bartuca et al. [20] reviewed the beneficial effect of a range of biostimulants on the phytoremediation performance of various crops. Enhanced availability of N may well function as a beneficial factor in this respect. However, to the best of our knowledge such investigation concerning any cationic toxic metal has not been widely studied and reported before in the specialized literature. The hypothesis tested is that in the presence of a plant growth parameter, such as increased soil N, chemical toxicity stress induced to purslane by Pb will be alleviated. The aim of this work was to test purslane as a potential phytoremediation species in soil spiked with Pb gradually up to very high concentrations in order to evaluate the tolerance of the plant and its behavior towards Pb exposure. Moreover, the addition of surplus N was tested as a factor that would boost plant vigor and thus have a beneficial effect on the plant’s response to Pb toxicity stress.

## 2. Materials and Methods

### 2.1. Soil Additions and Experimental Design

A pot experiment was established with soil obtained from the University of Thessaly Farm (Velestino at 39.394930 N, 22.757112 E), central Greece. The soil was pristine (total Pb content of 12.19 mg kg^−1^) with pH 7.8, CaCO_3_ = 10.4%, organic C = 1.5%, sand = 45%, and clay = 16% (all measured according to established methodology as per Koutroubas et al. [21]). We prepared a Pb spiking solution containing 10,000 mg Pb L^−1^ by dissolving 15.70 g (CH_3_COO)_2_Pb L^−1^. With this solution we spiked the soil at 5 gradual Pb dosages, as follows: (a) Control (no addition—hereafter referred to as Pb(0)); (b) Pb(150): Pb at 150 mg kg^−1^ soil by adding 15 mL of the spiking solution to 1 kg of soil; (c) Pb(300): Pb at 300 mg kg^−1^ soil by adding 30 mL of the spiking solution; (d) Pb(600): Pb at 600 mg kg^−1^ soil by adding 60 mL of the spiking solution; and (e) Pb(900): Pb at 900 mg kg^−1^ soil by adding 90 mL of the spiking solution. These treatments were replicated 20 times each. To half of the replicates, we added N at a rate equivalent to 300 kg N ha^−1^, i.e., 7 mg N kg^−1^ soil. This was added with 20 mL of a solution containing 14.3 g NH_4_NO_3_ L^−1^. The other half of the replicates were not amended with N. This resulted in 10 different treatments, each replicated 10 times: Pb(0) without N—hereafter referred to as Pb(0)N(0), Pb(0) with added N—Pb(0)N(1), Pb(150)N(0) (added Pb at 150 mg kg^−1^ without N), Pb(150)N(1) (Pb 150 mg kg^−1^ with added N), and, similarly, Pb(300)N(0), Pb(300)N(1), Pb(600)N(0), Pb(600)N(1), Pb(900)N(0), and Pb(900)N(1). Overall, the experimental design was that of two factors, namely Pb addition (at 5 rates) and N amendment (at two rates), resulting in a total of 100 pots: 5 Pb rates × 2 N rates × 10 replicates. The treated soils, 1 kg of dry matter each, were initially placed in 100 plastic bags and watered at 65% of their water-holding capacity. The soils were left for one month to equilibrate. During this time bags were kept open to avoid anoxic conditions, watered every few days according to their weight loss due to evaporation, and stirred thoroughly at regular intervals so that Pb and N could be incorporated as evenly as possible. During this time, purslane seeds were sown in plastic seed trays with moist soil, the same as that used for the experiment. After one month of incubation, the soil was placed in 2 L pots and young purslane plants were transferred to these pots, two plants per pot. After a few days, the plants were thinned to one per pot. The day of transplanting was considered to be the day of the commencement of the experiment (20 October 2019). Pots were placed according to a completely randomized design (CRD) into an unheated greenhouse, and watered regularly according to their needs so that they could retain their moisture level constant, while pot positions were regularly exchanged in order to compensate for any differences in light and temperature within the greenhouse. The experiment had a duration of 50 days (until 10 December 2019).

### 2.2. Sampling, Sample Processing, Analyses and Measurements

One week prior to the harvest date, we measured plant height (cm), and photosynthetic rate (µmol CO_2_ m^−2^ s^−1^) at a constant light intensity of 250 µmol cm^−2^ s^−1^ using the LI-Cor LI-6400XT Portable Photosynthesis System (LI-Cor, Lincoln, NE, USA)), whereas chlorophyll content (SPAD index) was measured using the OPTI-SCIENCES CCM-200 plus chlorophyll content meter (Opti-sciences, Hudson, NH, USA). Moreover, we measured the leaf area with the use of LI-3100C Area Meter (LI-COR Biosciences; Hellamco S.A., Athens, Greece) (all plant measurements are described in detail by [13,22]. At the end of the experiment, the aerial plant biomass was cut 2 cm above the soil surface and separated into leaves and stems. After that, plant parts were washed with distilled water and placed into paper bags. Subsequently, soil from the pots was sampled: We obtained 3 cores of soil made with a 5-cm-diameter plastic tube that was inserted into the pots at 3 random places. The sampled soil from each pot was thoroughly mixed, placed into paper bags, and taken for air drying. After being air-dried, soil samples were sieved through a 2 mm sieve. Then, roots were sampled by washing and removing carefully all adhered soil particles. Roots were also placed into paper bags. The three bags per pot containing plant parts (leaves, stems and roots), were then placed into an oven at 70 °C until no further weight loss was recorded and the dry weight of the plant parts was recorded. Then the aerial parts per pot were mixed, and both aerial biomass and roots were ground to a fine powder with a non-metallic rotary mill. For the analyses, 1.00 g of the plant biomass was weighed into porcelain crucibles, dry-ashed at 500 °C for 5 h, and extracted with 20 mL 20% HCl into 50 mL volumetric flasks, which were filled up to the mark with distilled H_2_O. Extracts were then analyzed for Pb with a flame atomic absorption spectrophotometer (Perkin Elmer A3030). As for the soil, after being air-dried, it was extracted for the available concentrations of Pb with DTPA (diethylene-triamine penta-acetate)-CaCl_2_-TEA (tria-ethyl-amine) at a ratio of 1:2 soil-to-solution. Moreover, the soil was extracted with aqua regia for the pseudo-total Pb concentrations (digestion at 130 °C for 3 h in a mixture of concentrated acids of HCl and HNO_3_ at a ratio of 3:1). All soil extracts (DTPA and aqua regia) were analyzed for Pb as in the case of the plant extracts. The limit of quantification of the Pb analysis was 100 μg L^−1^. Soil samples were also analyzed for residual N in the form of NO_3_-N: extraction was performed with 2 M KCl and the extracts were analyzed in a UV spectrophotometer as the difference in absorbance at 210 nm and 270 nm.

We also calculated some secondary indices based on the primary laboratory data, as follows:

     Bioavailability index, BAI (unitless) = Pb concentration in plant (in mg kg^−1^ plant)/DTPA-extractable Pb concentration in soil (in mg kg^−1^ soil) Translocation factor, TF (unitless) = Pb in aerial biomass (in mg kg^−1^)/Pb concentration in roots (in mg kg^−1^)   Pb uptake by plant (mg Pb in plant per pot) = Pb concentration in plant (in mg Pb kg^−1^ plant) × dry biomass (g pot^−1^)/1000 Harvests needed to reduce soil Pb to half (H_1/2_) = (Pseudo-total concentration in soil × 0.5)/Pb uptake by plant

### 2.3. Quality Control and Statistical Interpretation

For data quality assurance, all extraction batches included a blank sample in order to avoid any bias due to laboratory contamination, and all analyses were performed in triplicates. Results were considered acceptable when the relative standard deviation was <10%. All data were analyzed for a two-way ANOVA by IBM SPSS Statistics 26 after the results complied with the normality test, with the first factor being Pb addition and the second one the added N. When significant effects were recorded, Duncan’s post-hoc analysis was performed at *p* < 0.05 for means comparison.

## 3. Results

### 3.1. Soil Pb

Added Pb to soil was estimated with a pseudo-total (aqua regia) extraction, in order to examine how much Pb was really found in soil after its amendment. Unamended control treatment contained a small concentration of Pb (12.2 mg kg^−1^), and the actual concentrations recorded at the added levels of 150, 300, 600, and 900 mg kg^−1^ were 145.7, 281.6, 585.6, and 862.3 mg kg^−1^, respectively (Table 1). As for the plant response to added Pb, plant height increased significantly with added N in any Pb addition (the *p*-value of the factor “added N” equaled 0.001; Table 2). It is noteworthy that there was a slight increase in recorded plant height even with added Pb within the same N regime, with Pb(900)N(0) being 29.97 cm vs. 24.84 cm in Pb(0)N(0) at no added N, and 31.84 cm (Pb(900)N(1)) vs. 28.93 cm (Pb(0)N(1)) at added N.

**Figure 1:**
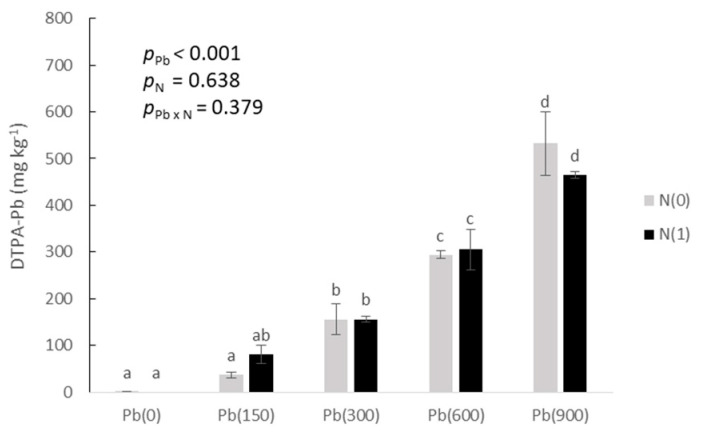
Pb concentration in soil extracted with DTPA as affected by the addition of Pb (0, 150, 300, 600, and 900 mg kg^−1^ soil) and with (N1) and without added nitrogen (N0). Different letters above each bar denote significantly different treatments at *p* < 0.05.

As for the DTPA extractions (Figure 1), Pb concentration increased from the non-detected levels at Pb(0) to 532.6 mg kg^−1^ at Pb(900)N(0) and 464.1 mg kg^−1^ at Pb(900)N(1), with the two treatments having no significant differences.

### 3.2. Plant Growth Parameters

Similar were the trends in dry matter content of leaves, stems, and roots: they seem to have been highly affected by the factor “added N” (*p* < 0.001 for the aerial parts; *p* = 0.011 for roots), and also to have an increasing trend with added Pb, regardless of the N regime. However, the leaf area (in cm^2^) was only affected by N fertilization, whereas with added Pb there seemed to be no differences at Pb(900) compared to Pb(0) both in the treatment with and without added N.

### 3.3. Plant Physiological Parameters

**Figure 2:**
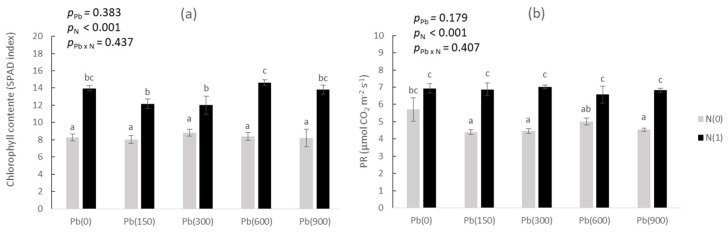
(**a**) Chlorophyll content index (OPTI) and (**b**) photosynthetic rate (LICOR) in purslane as affected by the addition of Pb (0, 150, 300, 600, and 900 mg kg^−1^ soil) and with (N1) and without added nitrogen (N0). Different letters above each bar denote significantly different treatments at *p* < 0.05.

Apart from these growth parameters, we measured plant physiological indices, i.e., chlorophyll content index (CCI; Figure 2a) and the photosynthetic rate at a light intensity of 250 μmol (Figure 2b). Data suggest that added Pb did not have any significant effect on CCI (*p*_Pb_ = 0.383), while it was affected by added N (*p*_N_ < 0.001), with all differences within each added N regime being rather small. Similar were the effects on the plant’s photosynthetic rate, with the difference being that at Pb(900)N(0) the rate decreased significantly compared to Pb(0)N(0). In the added N treatments, no such effect was observed between Pb(900) and Pb(0)—no significant differences were recorded. The overall trend in both parameters is the increase in the rate of the physiological functions in the treatments of the added N compared to those without added N.

### 3.4. Content of Pb in Plant

**Figure 3:**
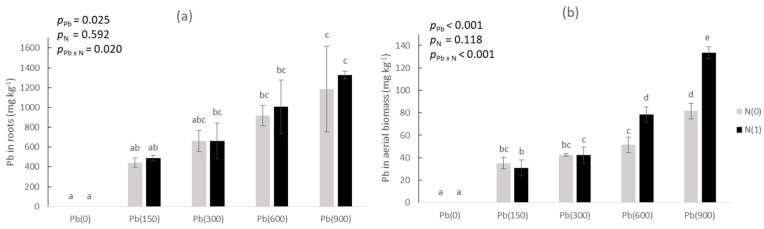
Pb concentrations (**a**) in roots, and (**b**) in aerial biomass in purslane as affected by the addition of Pb (0, 150, 300, 600, and 900 mg kg^−1^ soil) and with (N1) and without added nitrogen (N0). Different letters denote significantly different treatments at *p* < 0.05.

With added Pb, the metal concentration increased in plant roots (Figure 3a), and aerial biomass (Figure 3b). In roots (Figure 3a), at Pb(0) extracted concentrations were not detected, and gradually increased at Pb(900)N(0) to 1184 and 1326 mg kg^−1^ ((Pb(900)N(1)—but there was no significant difference between the two treatments). Added N did not affect Pb absorption by roots (i.e., there was no significant effect in the “added N” factor, with *p*_N_ = 0.592). As for Pb in aerial biomass (Figure 3b), it did increase significantly with added Pb gradually from Pb(0) (where it was not detected) to Pb(900); however, between Pb(900)N(0) and Pb(900)N(1) there was a significant difference: 81.5 mg kg^−1^ vs. 133.5 mg kg^−1^. Similarly, there was a significant difference between Pb(600)N(0) (51.5 mg kg^−1^) and Pb(600)N(1) (78.5 mg kg^−1^).

### 3.5. Transfer Indices and Plant Uptake of Pb

**Figure 4:**
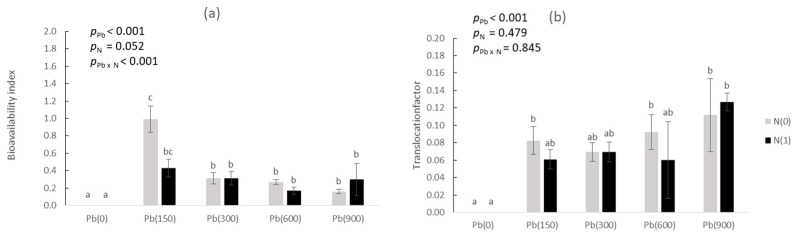
(**a**) Bioavailability index (BAI = Pb in DTPA extractions over Pb in aerial biomass) and, (**b**) translocation factor (TF = Pb in aerial biomass over Pb in roots) in purslane as affected by the addition of Pb (0, 150, 300, 600, and 900 mg kg^−1^ soil) and with (N1) and without added nitrogen (N0). Different letters above each bar denote significantly different treatments at *p* < 0.05.

Concerning the soil-to-plant transfer index (bioavailability index, BAI; Figure 4a), we found that it decreased with added Pb from Pb(150) to Pb(900): At Pb(150)N(0) it was 0.99 and at Pb(150)N(1) 0.43 (with no significant difference between the two); these values decreased significantly to 0.16 at Pb(900)N(0) and to 0.30 at Pb(900)N(1) (similarly, no differences between the two). As for the root-to-shoot translocation factor (Figure 4b), although there was a slightly increasing trend with added Pb, there was no significant difference at Pb(900) compared to Pb(150) either at N(0) or at N(1).

As for Pb uptake, we found that it was both Pb and N dependent in roots and in aerial biomass uptake alike (Table 3). Root uptake increased from 0 to 1.65 mg Pb pot^−1^ at N0 and to 3.24 mg Pb pot^−1^ at N1. Aerial biomass uptake was the highest at Pb(900)N(0): 0.17 mg Pb pot^−1^ and 0.51 mg Pb pot^−1^ at Pb(900)N(1), with differences between the two N regimes being statistically significant at *p* < 0.001. 

With uptake, the number of harvests necessary to reduce to half the initial soil Pb levels can be calculated (Table 4). From these calculations, the Pb(0) controls were excluded due to the fact that there was no uptake (Pb concentrations were zero). We found that added N resulted in a decreased number of necessary harvests, but the difference was significant only at Pb(900). The number of harvests ranged from 120 at Pb(150)N(0) to 307 at Pb(900)N(0) in the no added N regime, and from 76 at Pb(150)N(1) to 131 at Pb(900)N(1) in the added N treatments.

**Figure 5:**
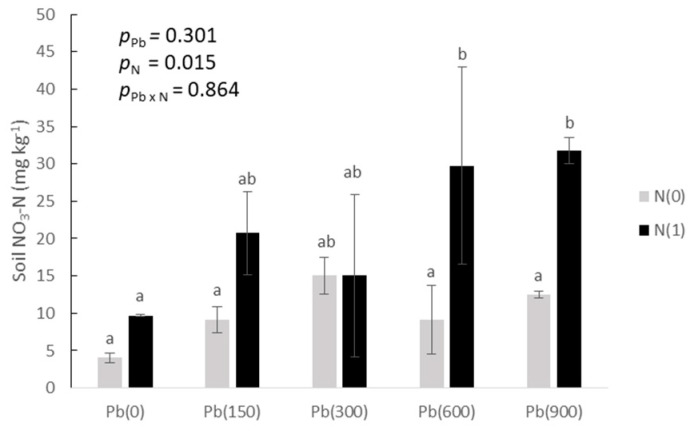
Residual N (as NO_3_-N) in soil cultivated with purslane as affected by the addition of Pb (0, 150, 300, 600, and 900 mg kg^−1^ soil) and with (N1) and without added nitrogen (N0). Different letters above each bar denote significantly different treatments at *p* < 0.05.

Added N resulted in residual soil N, which was calculated as NO_3_-N at the end of the plant growth (Figure 5). Residual N concentrations were significantly higher in the added N treatments at Pb(150), Pb(600), and Pb(900). Residual N did not seem to have a significant increase with added Pb levels except for the control in the added N treatments.

## 4. Discussion

In this work, we estimated pseudo-total Pb in order to examine whether added Pb would be recovered. Table 1 shows that Pb recovery was rather successful, and this is associated with the fact that we chose to incubate and thoroughly mix our soil in the preparation stages of the experiment. As a result, recovery ranged from 94% to 98%. It was rather unexpected the fact that added Pb did not seem to have a detrimental effect on plant growth parameters, recorded in Table 2. Comparing the Pb treatments within the same N regime, plant height, and the dry weight of the aerial parts (leaves and stems) seemed to increase with added Pb doses; this resulted in the growth parameters having higher values at Pb(900) than at Pb(0), both in the added N and in the no-added-N regimes. The reason for this unexpected finding cannot be attributed to any non-controlled beneficial effect in the experiment, because growth conditions (e.g., light and temperature) were the same for all pots. This can likely be attributed to acetate, which was added along with Pb. Acetate has recently been used as a biostimulant assisting the growth of various plants against drought [23]. Although this effect is not conclusive (as Pb-induced stress is different from that of reduced water supply), the promoted purslane growth can well have benefited from the presence of acetate. This is an indication of the fact that Pb toxicity stress was rather masked by the beneficial effect of added acetate. This finding is novel and noteworthy given the fact that the dose of acetate in soil was rather minuscule: at the high Pb addition of Pb = 900 mg kg^−1^, added acetate was 513 mg kg^−1^ soil, equal to 4.35 mmol kg^−1^ soil. As a plant growth-promoting agent, acetate functions at added concentrations of an order of magnitude higher than that, and also it is applied as a solution directly to plants—intervening soil always considerably decelerates either beneficial or toxicity agents added to it. For example, Rahman et al. [24] added up to 30 mmol L^−1^ acetic acid as a foliar spray to mung beans (*Vigna radiata*). This, seen from a different perspective, can be an indication that added Pb has not had a dramatic toxic effect on the test plant—a finding likely exhibiting its behavior as a tolerant species towards abiotic stress. Moreover, all plant growth parameters benefited significantly from the presence of N; at any given added Pb rate, values of the parameters with N were significantly higher than those without N, especially in the high Pb additions. This was the case with all studied growth parameters, i.e., height, dry weight of aerial parts (leaves and stems), roots, and leaf area. This is in agreement with Thalassinos et al. [14], who reported the same beneficial effect of added N concerning Cr(VI)-stressed purslane plants. It is known that in the presence of a certain stress, plants’ tolerance towards another stress decreases. Here we tested the hypothesis that an agent that promotes growth and benefits plant physiological functions would mitigate Pb toxicity. Our findings concerning the plant growth parameters seem to indicate that growth even increased instead of being decreased more conservatively, as initially expected. 

The physiology measurements (Figure 2) largely confirmed the above-stated findings: Added N improved both CCI and the photosynthetic rate which increased significantly at N1 compared to N0, a finding exhibiting the beneficial effect of added N over the photosynthetic capability of the test plant [25]. However, contrary to the growth findings, no benefit seemed to be established with added Pb—the low additions of acetate along with Pb did not have any effect on the physiological parameters. On the other hand, neither did the extremely high levels of added Pb. Such a finding indicates the tolerance that the test plant exhibits towards Pb stress, in line with the findings for other plants (*Chrysopogon zizanioides*—[26]; *Tagetes erecta*—[27]; soybean—[28]).

The non-affected growth and non-decreased physiological functions of purslane were recorded despite the fact that Pb concentrations absorbed by the plant were very high (Figure 3), higher than those reported elsewhere [29,30,31]. It is noteworthy that Pb in purslane aerial biomass increased significantly between Pb(600)N(0) and Pb(600)N(1). Similarly, there was a significant increase between Pb(900)N(0) and Pb(900)N(1). The enhanced plant Pb concentration within the same added Pb treatment could have been caused by added N. It is known that added N in the form of ammonium is readily nitrified—a biological oxidation process that results in the elution of 2 moles of H^+^ in the soil solution per mol NO_3_-N produced, and thus leads to temporal acidification. This effect could have triggered this increase within the same Pb additions, due to the fact that acidification increases the availability of cationic metal species such as Pb^2+^.

The fact that Pb was spiked in the soil, a condition that encourages higher absorption by a test plant, could explain the relatively high Pb concentrations we found for purslane. The levels of Pb in roots surpassed the threshold required by a plant to be categorized as a hyperaccumulator, e.g., 1000 mg kg^−1^ of dry matter plant [32,33], although this is a non-harvested plant part. Aerial biomass absorbed high concentrations of Pb, which, as in the case of the roots, were rate-dependent. It is noteworthy that making comparisons with the maximum threshold limits set up by the 2006/1881/EU Directive [34], “leaf vegetables” (category 3.1.10) should not surpass Pb = 0.10 mg kg^−1^ “wet weight,” i.e., ca. 1.0 mg kg^−1^ dry weight plant. This threshold was surpassed in our experiment by purslane even at Pb(150), while at Pb(900)N(1) the Pb content was 133-fold higher than the threshold, making our test plant totally unsuitable for consumption in case it was cultivated in severely Pb-contaminated soils. The content of Pb in purslane (and in any other plant grown in non-contaminated soils) is not expected to exceed the level of a few hundred μg kg^−1^ dry weight (as was reported by Bagdatlioglu et al. [35] who found Pb = 0.35 mg kg^−1^). This finding eliminates any possibility of utilizing purslane as an excluder species to be cultivated in contaminated soils—plants that would withstand high pollutant levels by excluding them from uptake.

Further testing the possibility of categorizing purslane as a potential Pb accumulator, we calculated BAI (Figure 4a) and TF (Figure 4b). As for BAI, the requirement is that the value must be as high as possible and close to 1.0, while for TF that the value must exceed 1.0. BAI reached a value > 1.0 only in the lowest addition of Pb, and was subsequently reduced with increasing Pb rate, a finding in accordance with Levizou et al. [36], who reported that it should be expected that the assimilation rate of any plant for a toxic element decreases as soil concentrations increase, a trend that leads to decreasing BAI as soil concentrations increase. This was also agreed upon by Antoniadis et al. [33]. Similar to our findings, other works also report a BAI of a lot less than 1.0 in purslane grown in Pb-contaminated soils. Osma et al. [37] found a concentration in plants ranging from 4 to 6 mg kg^−1^ in soils with Pb up to 70 mg kg^−1^—a BAI of ca. 0.1. A similar BAI value was found by Naz et al. [38] who reported Pb in purslane up to 45 mg kg^−1^ in soil spiked with up to 500 mg Pb kg^−1^. As for the TF, although values were much lower than 1.0, it must be stated that the TF > 1.0 requirement is seldom achieved by plants grown in soil [39,40,41,42].

It is noteworthy, however, that due to the considerable increase in Pb concentration with added Pb, which was accompanied by a slight (but significant) increase in biomass yield, Pb uptake (the product of the multiplication of the two above-stated factors) increased significantly with added Pb, especially in the treatments with the added N (Table 3). This is a major finding that seems to confirm our hypothesis—purslane’s capability as a potential Pb phytoremediation species was boosted when growth conditions were improved (here, by adding N, which increased growth and physiological functions). This was also confirmed by the fact that the number of purslane harvests necessary to decrease Pb soil levels down to half the initial concentration (an index of the phytoremediation robustness of any given potential accumulator plant) was in the order of magnitude of a few hundred, a number that decreased to 131 at Pb(900)N(1) (Table 4). This constitutes a reasonable number of harvests when compared to other literature reports, where such a number is usually much higher [42], especially when considering the short growth cycle of the species and the possibility to perform more than one growth cycle per growing season, depending on the growing conditions. The reason is explained by the fact that Pb is usually a rather immobile element in soil, and thus its expected transfer from soil to plant is rather slow—thus soil decontamination by plants requires a number of consecutive harvests which are orders of magnitude higher than those reported here. Although this analysis is valuable to showcase the parameter of the time required for the phytoremediation process, we acknowledge some shortcomings that should be taken into consideration: (a) the number of harvests assumes that purslane retains the same yield over time, implying continuous high N inputs; (b) the rate of Pb uptake remains the same—although it is expected that over time Pb becomes gradually more immobile in the soil, as also discussed earlier. Thus, the extrapolation from pot- to field-based results should not be performed without due caution.

## 5. Conclusions

- Added Pb did not cause any adverse effects on purslane growth (height, dry biomass of leaves and branches, as well as of roots, and leaf area), neither did it affect physiological parameters (chlorophyll content index and photosynthetic rate), a notable finding exhibiting the tolerance of purslane towards Pb stress. Added N boosted the plant’s growth and physiological functions, but also acetate may have played a role in this effect.
- Pb concentration to plant increased significantly with added soil Pb, and its uptake was also positively affected by added N. This indicates that a tolerant plant with the potential to be used for soil decontamination increases its uptake capability when growth conditions are promoted and when plant vigor is improved—a finding that confirmed our hypothesis.
- Although purslane shows great potential as a phytoremediation species, as also indicated by the relatively low number of necessary harvests to half initial soil Pb levels, more research is necessary, especially under real field conditions in soils contaminated over long periods of time—situations under which uptake is expected to be slower and Pb mobility more conservative.

## Figures and Tables

**Table 1:** Added and measured Pb with aqua regia (pseudo-total) (units in mg Pb kg^−1^ soil). Standard deviations of measured Pb are shown in parentheses.

Treatment	Pb Added	Pb Measured
Pb(0)	0	12.2 (0.91) a
Pb(150)	150	145.7 (19.8) ab
Pb(300)	300	281.6 (15.2) b
Pb(600)	600	585.6 (72.9) c
Pb(900)	900	862.3 (63.6) d

Different letters in the same column indicate significant differences at *p* < 0.05.

**Table 2:** Plant parameters in the purslane cultivated in soil with added Pb with and without N.

		Height (cm)	Leaf (g DM)	Branches (g DM)	Total (g DM)	Root (g DM)	Leaf Area (cm^2^)
No added N	Pb(0)	24.84 a	0.25 a	0.79 a	1.04 a	0.35 a	178.0 a
	Pb(150)	28.99 b	0.53 b	1.22 bc	1.75 b	0.34 a	210.3 a
	Pb(300)	30.53 c	0.56 b	1.24 bc	1.80 b	0.35 a	160.4 a
	Pb(600)	28.74 b	0.60 b	1.05 ab	1.65 b	0.25 a	222.0 a
	Pb(900)	29.97 bc	0.48 b	1.24 bc	1.72 b	0.41 a	230.5 a
Added N	Pb(0)	28.93 b	0.92 c	1.76 de	2.68 c	0.52 ab	494.3 c
	Pb(150)	29.01 b	1.06 cde	2.15 f	3.22 d	0.56 ab	465.3 bc
	Pb(300)	30.53 c	0.56 cd	1.24 ef	1.80 cd	0.35 a	160.4 bc
	Pb(600)	30.88 c	1.18 de	1.43 cd	2.62 c	0.34 a	400.3 b
	Pb(900)	31.84 c	1.24 e	2.57 g	3.81 e	0.80 b	492.0 c
	Significance						
	*p*_Pb_	<0.001 ***	<0.001 ***	<0.001 ***	<0.001 ***	0.015 *	0.121 ^NS^
	*p*_N_	0.001 **	<0.001 ***	<0.001 ***	<0.001 ***	0.011 *	<0.001 ***
	*p*_PbxN_	0.334 ^NS^	0.233 ^NS^	0.004 **	0.018 *	0.178 ^NS^	0.170 ^NS^

Different letters within columns indicate significant differences at *p* < 0.05. NS = non-significant. DM = dry matter. * Significant at *p* < 0.05. ** Significant at *p* < 0.01. *** Significant at *p* < 0.001.

**Table 3:** Uptake of Pb by purslane (in mg of Pb in plant per pot).

		Pb Uptake in Root	Pb Uptake in Aerial Biomass
No added N	Pb(0)	0.00 a	0.00 a
	Pb(150)	0.56 ab	0.07 ab
	Pb(300)	0.85 ab	0.08 ab
	Pb(600)	1.09 ab	0.14 ab
	Pb(900)	1.65 b	0.17 b
Added N	Pb(0)	0.00 a	0.00 a
	Pb(150)	0.94 ab	0.09 ab
	Pb(300)	0.85 b	0.08 b
	Pb(600)	1.68 b	0.15 b
	Pb(900)	3.24 c	0.51 c
	Significance		
	*p*_Pb_	<0.001 ***	<0.001 ***
	*p*_N_	0.010 **	0.001 **
	*p*_PbxN_	0.355 ^NS^	<0.001 ***

Different letters within columns indicate significant differences at *p* < 0.05. NS = non-significant. ** Significant at *p* < 0.01. *** Significant at *p* < 0.001.

**Table 4:** Number of harvests of purslane necessary to halve the initial Pb soil concentration.

	No Added N	Added N	Significance
Pb(0)	—	—	—
Pb(150)	120 ab	76 a	NS
Pb(300)	183 abc	183 abc	NS
Pb(600)	244 bc	230 abc	NS
Pb(900)	307 c	131 ab	*

Different letters within columns indicate significant differences at *p* < 0.05. * Significant at *p* < 0.05.

## Data Availability

Data is contained within the article.
